# Genetic subtyping by Whole Exome Sequencing across Diffuse Large B Cell Lymphoma and Plasmablastic Lymphoma

**DOI:** 10.1371/journal.pone.0318689

**Published:** 2025-03-11

**Authors:** Aitana Avendaño Pomares, Laura Rodríguez Merino, Sonia González, Jordi Morata, Raúl Tonda, Patricia Arribas, José Revert, Estrella Carrillo, Carlos Grande, Josep Maria Roncero, Jaime Pérez de Oteyza, Concepción Nicolás, Norma Gutierrez, Pau Abrisqueta, Antonio Gutiérrez, Ángel Ramírez-Páyer, Alejandro Martin Garcia-Sancho, Eva González-Barca, Santiago Montes-Moreno, on behalf of GELTAMO group (Grupo Español de Linfomas y Trasplante Autólogo de Medula Osea), Spain

**Affiliations:** 1 Translational Hematopathology and Anatomic Pathology Department, Valdecilla/IDIVAL, UNICAN, Santander, Spain; 2 Hematology Department, Valdecilla/IDIVAL, Santander, Spain; 3 Centre Nacional d’Anàlisi Genòmica, Barcelona, Spain; 4 Hematology Department, Hospital Virgen del Rocío, Seville, Spain; 5 Hematology Department, Hospital Universitario 12 de octubre, Madrid, Spain; 6 Hematology Department, ICO Girona, Girona, Spain; 7 Hematology Department, CIOCC, Madrid, Spain; 8 Hematology Department, Hospital Central de Asturias, Oviedo, Spain; 9 Hematology Department, Hospital Universitario de Salamanca, IBSAL, CIBERONC, University of Salamanca (USAL), Salamanca, Spain; 10 Hematology Department, Hospital Universitario Vall d’Hebron, Barcelona, Spain; 11 Hematology Department, Hospital Universitario Son Espases, Palma de Mallorca, Spain; 12 Insititut Català d’Oncologia Hospitalet, Universitat de Barcelona, Insititut d’Investigació Bellvitge (IDIBELL), Barcelona, Spain; European Institute of Oncology, ITALY

## Abstract

Diffuse Large B-Cell Lymphoma (DLBCL) is a heterogeneous disease characterized by a limited number of molecularly defined subtypes. Recently, genomic-based algorithms have been proposed for the classification of this disease. The whole exome sequencing was conducted on 108 diagnostic samples of diffuse large B-cell lymphoma (DLBCL). Somatic variants, predicted copy number alterations (CNAs), and available fusion data were utilized to classify the cases. Additionally, the enrichment of mutations in the TP53, MYC, and MAPK/ERK pathways was analyzed. Genetic subtypes were identified in approximately 55% of the cases. Cases with a specific genetic subtype exhibited a significantly higher Tumor Mutation Burden compared to molecularly unclassified cases (Mann-Whitney U test, p =  0.024). The prevalence of subtypes varied according to the cell of origin phenotypes. GC-B type DLBCL NOS were classified as EZB (5 cases, 16%), ST2 (5 cases, 16%), and BN2 (1 case, 3%). Four cases (13%) were genetically composite. Three cases of HGBCL/DLBCL double-hit (MYC & BCL2) were classified as EZB-MYC. Forty-three non-GC-B type DLBCL cases were classified as ST2 (5 cases, 11%), BN2 (6 cases, 14%), and MCD (3 cases, 7%). Nine cases were genetically composite (20%). MYC pathway mutations were enriched in cases with EZB and ST2 genetic features, while they were absent in the MCD subtype. TP53 mutations were identified in 11% of the cases. Plasmablastic lymphomas exhibit genetic diversity, with 27% of tumors classified as ST2. Recurrent somatic mutations indicate dysregulation of the JAK/STAT, MAPK/ERK, and tyrosine kinase signaling pathways.

## Introduction

Diffuse Large B-Cell Lymphoma (DLBCL) is a heterogeneous disease characterized by a limited number of molecularly defined subtypes compared to other hematolymphoid neoplasms [1].

The application of high-throughput technologies, such as Next Generation Sequencing (NGS) and Copy Number Alterations (CNAs), has led to the development of at least two classification algorithms that integrate somatic mutations, CNAs, and fusions. These include the NCI classifier and the consensus cluster classification [2,3]. However, the feasibility of applying these complex methods to routine molecular diagnosis remains uncertain. To address this issue, targeted NGS algorithms have been developed, including the HMRN algorithm [4,5] and others [6].

Recently, genomic-based algorithms have become available for the classification of diffuse large B-cell lymphoma (DLBCL), including the LymphGen tool [7]. To date, a single large retrospective registry-based analysis utilizing targeted sequencing, without copy number alterations (CNAs) data, has produced consistent results with the National Cancer Institute (NCI) classifier [4] and has validated the prognostic impact of specific genetic subtypes (i.e., N1, EZB-MYC) in R-CHOP-treated DLBCL patients. Importantly, the identification of specific genetic events, such as the MYC expression signature and MYC mutations, has allowed for improved granularity in the classification of genetic subgroups, such as EZB-MYC, which may influence clinical decision-making. However, 50% of DLBCL cases remain unclassified (molecular NOS) using both the LymphGen and modified HMRN classifiers [4].

The genetic subtyping of specific entities of diffuse large B-cell lymphoma, as classified by the current WHO guidelines [1], has not been thoroughly addressed using the Lymphgen classifier. In this context, a clear association has been established between extranodal large B-cell lymphomas that occur in immune-privileged sites and the MCD genetic signature [7]. Furthermore, the integration of mutational data and gene expression characteristics facilitates the identification of EZB mutated-MYC active tumors, which include Dual Hit (DH) large B-cell lymphomas— characterized by concurrent MYC and BCL2 translocations—or tumors exhibiting high MHG gene expression signatures [4,8,9].

Other large B cell lymphoma entities, such as plasmablastic lymphoma, have been found to carry mutations in the JAK/STAT pathway, as well as in the MAPK/ERK and NOTCH pathways [10–12], and exhibit expression programs associated with terminal B cell differentiation [13]. The application of genetic classification algorithms to this entity may facilitate the identification of potential avenues for targeted therapy and uncover further genetic granularity in the disease.

Here, we aimed to utilize the LymphGen tool for disease classification by analyzing somatic mutation data and predicted copy number alterations (CNAs) obtained from whole exome sequencing (WES). This study involved a cohort of 108 large B cell lymphoma cases sourced from GELTAMO clinical trials and a retrospective multicentric series. Our objective was to validate the tool’s applicability across various large B cell lymphoma subtypes, including not only Diffuse Large B-Cell Lymphoma (DLBCL) Not Otherwise Specified (NOS) but also High Grade/DLCBL DH/NOS and plasmablastic lymphoma cases.

## Results

### Prevalence of genetic subgroups among DLBCL entities. DLBCL NOS, HGBCL DH/NOS and other rare DLBCL entities

The 108 cases were classified into various entities of large B-cell lymphoma according to the updated WHO classification [1]. This included 75 cases of diffuse large B-cell lymphoma not otherwise specified (DLBCL NOS), 22 cases of plasmablastic lymphoma, 3 cases of T-cell histiocyte-rich large B-cell lymphoma (TCHRBCL), 3 cases of high-grade B-cell lymphoma/DLBCL double-hit (MYC and BCL2), 1 case of high-grade B-cell lymphoma not otherwise specified (BCL NOS), 1 case of gray zone DLBCL, 1 case of Epstein-Barr virus-positive DLBCL, 1 case of primary mediastinal B-cell lymphoma (PMBL), and 1 case of follicular lymphoma grade 3B/follicular large B cell lymphoma [1]. Additionally, one case diagnosed as Burkitt lymphoma was excluded.

**Fig 1:**
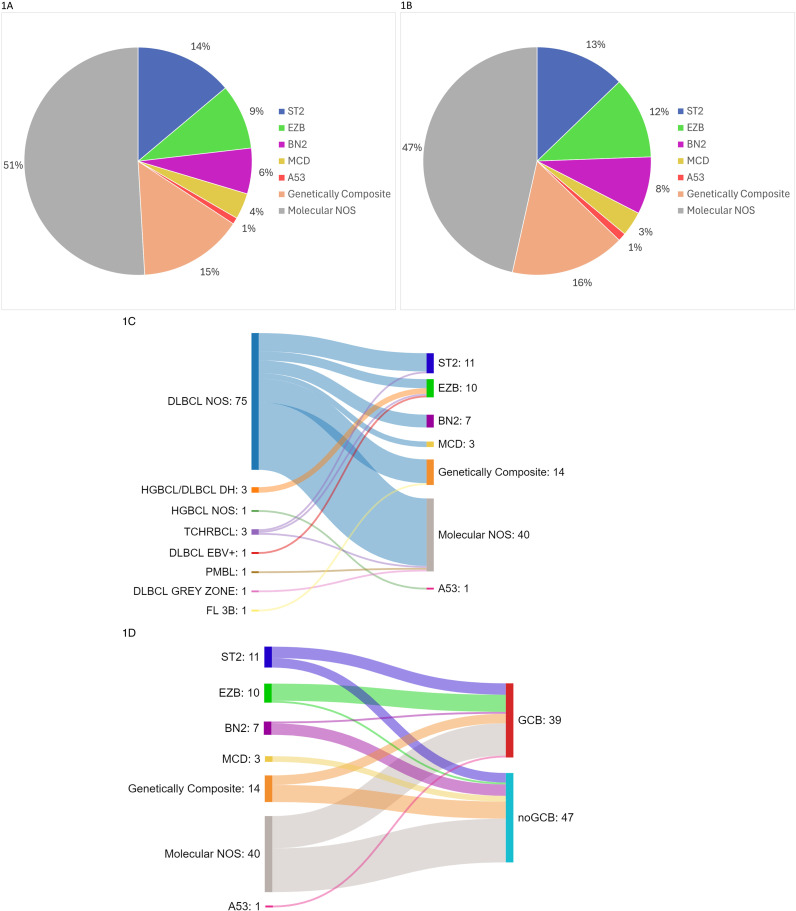
Genetic Classification by Lymphgen. A. Genetic classification by Lymphgen identified a specific subgroup in fifty-three cases (49%), while the remaining fifty-five cases were classified as unclassified (molecular subtype NOS/Other). B. DLBCL cases exhibited a similar distribution of genetic subtypes, with ST2 being the most prevalent subtype. C. A Sankey plot illustrating the genetic subtyping by Lymphgen across various large B-cell lymphoma entities. Notably, some entities, such as HGBCL/DLBCL DH, display homogeneous genetic features (i.e., EZB subtype). D. A Sankey plot depicting the genetic subtyping by Lymphgen according to cell of origin subtypes in DLBCL NOS cases. As shown, EZB is preferentially associated with GCB-type DLBCL, while both MCD and BN2 subtypes demonstrate a higher prevalence in non-GCB-type DLBCL tumors.

The two cases exhibiting concurrent MYC and BCL6 translocations without BCL2 rearrangements were categorized within the DLBCL NOS group, as recommended by the current WHO classification (1). Genetic classification using LymphGen 2.0 identified a specific genetic subtype in fifty-three cases (49%), while the remaining fifty-five cases were deemed unclassified (molecular subtype NOS/Other) (Fig 1).

Cell of origin classification based on immunohistochemistry (IHC) was successfully obtained in all 86 cases of diffuse large B-cell lymphoma (DLBCL). Among these, 39 cases (45%) were classified as germinal center B (GC-B) type, while 47 cases (55%) were identified as non-GC-B type. Focusing specifically on the DLBCL NOS category, 31 out of 75 cases were classified as GC-B type (41%), and 43 cases (59%) were classified as non-GC-B type. The LymphC2x-based classification was achieved in 13 cases, comprising 3 GC-B, 9 activated B-cell type (ABC), and 1 unclassified case. The concordance rate with IHC was 77%, with 10 out of 13 cases receiving equivalent subtype assignments. This concordance rate is slightly lower than previously published data, which may be attributed to the small sample size. A summary of the major pathological features of these patients is presented in S1 Table in S1 File.

### Tumor Mutation Burden across DLBCL subtypes. Prevalence of Genetic subtypes in 86 DLBCL cases

A total of 61036 somatic variants were identified in the 108 cases. 88.5% corresponded to missense mutations, 5.5% to nonsense mutations, 2.3% to splice site, 1.03% to frame-shift deletions, 1.11% to frame-shift insertions, 0.89% to in frame deletions, 0.35% to in-frame insertions, 0.13% to translation start site, 0.07% to non-stop mutations. Mean sequencing depth was 50.87 (12.94 SD). The median sequencing depth was 51.87. mad (Median Absolute Deviation) was 11.07. IQR (Interquartile Range) was 15.38.

After WES median Tumor Mutation Burden (TMB, i.e., absolute number of nonsynonymous variants/60 Mb exome coverage) in 108 cases was 13. Median TMB in 86 DLBCL cases was 17.

Interestingly, cases of DLBCL with a specific genetic subtype (see below) exhibited a significantly higher median TMB =  24) compared to molecular NOS cases (median TMB =  9), as determined by the Mann-Whitney U test (p =  0.024).

Genetic classification by LymphGen into a specific subgroup was achieved in forty-six cases (53%), while the remaining forty cases were unclassified (molecular subtype NOS/Other) (Fig 1).

A comprehensive description of the genetic subtype LymphGen features identified in each case can be found in S2 Fig and S3 Table in S1 File.

Of the DLBCL cases genetically classified by the LymphGen algorithm (i.e., genetic subtypes), eleven were categorized as ST2 (13%), ten as EZB (12%), seven as BN2 (8%), three as MCD (3%), and one as A53 (1%). Interestingly, fourteen cases (16%) were genetically composite, including seven ST2/EZB, two ST2/BN2, one ST2/MCD, and four triple/quadruple composites (ST2/EZB/MCD; ST2/EZB/BN2; ST2/BN2/MCD; and ST2/EZB/BN2/MCD) (Fig 1B). DLBCL NOS cases (n =  75) exhibited a similar prevalence of genetic subtypes, with ten classified as ST2 (13%), five as EZB (7%), seven as BN2 (9%), three as MCD (4%), thirteen as composite (17%), and thirty-seven as molecular NOS/Other (50%).

According to the cell of origin subtype based on IHC, 31 cases of GC-B type DLBCL NOS were classified as follows: EZB (5/31, 16%), ST2 (5/31, 16%), and BN2 (1/31, 3%). Four out of the 31 cases (13%) were genetically composite, while sixteen cases remained classified as molecular NOS (16/31, 52%). Additionally, 44 cases of non-GC-B type DLBCL were categorized as ST2 (5/44, 11%), BN2 (6/44, 14%), and MCD (3/44, 7%). Nine cases were genetically composite (9/44, 20%), and twenty-one cases remained classified as molecular NOS (21/44, 48%) (Fig 1D).

The study included ten cases of pure EZB genetic subtype diffuse large B-cell lymphoma, which comprised germinal center B-cell (GCB) type DLBCL NOS, high-grade/DLBCL double-hit (DH) lymphoma characterized by MYC and BCL2 rearrangements (three cases), as well as rare B germinal center-derived DLBCL subtypes, including T-cell/histiocyte-rich B-cell lymphoma. Notably, a single rare case of EBV-positive DLBCL with a non-GCB phenotype exhibited EZB genetic features. We identified three cases (30%) of the EZB-MYC subtype, defined by an EZB genetic profile and MYC translocation. All these cases were classified as high-grade B-cell lymphoma/DLBCL DH (MYC and BCL2) based on histopathological evaluation.

DLBCL NOS with concurrent MYC and BCL6 translocations (two cases) exhibited composite EZB/ST2 and NOS/Other features. The phenotypic characteristics were also diverse, with the composite EZB/ST2 case displaying a non-GCB phenotype (ABC type according to LymphC2x) and the Other/NOS case demonstrating a GCB phenotype. Consequently, the phenotypic and genetic features were homogeneous within the HGBCL/DLBCL DH (MYC&BCL2) category, characterized by a GCB phenotype and EZB-MYC genetic features. In contrast, MYC&BCL6 double-hit cases exhibited disparate genetic and phenotypic characteristics.

**Fig 2:**
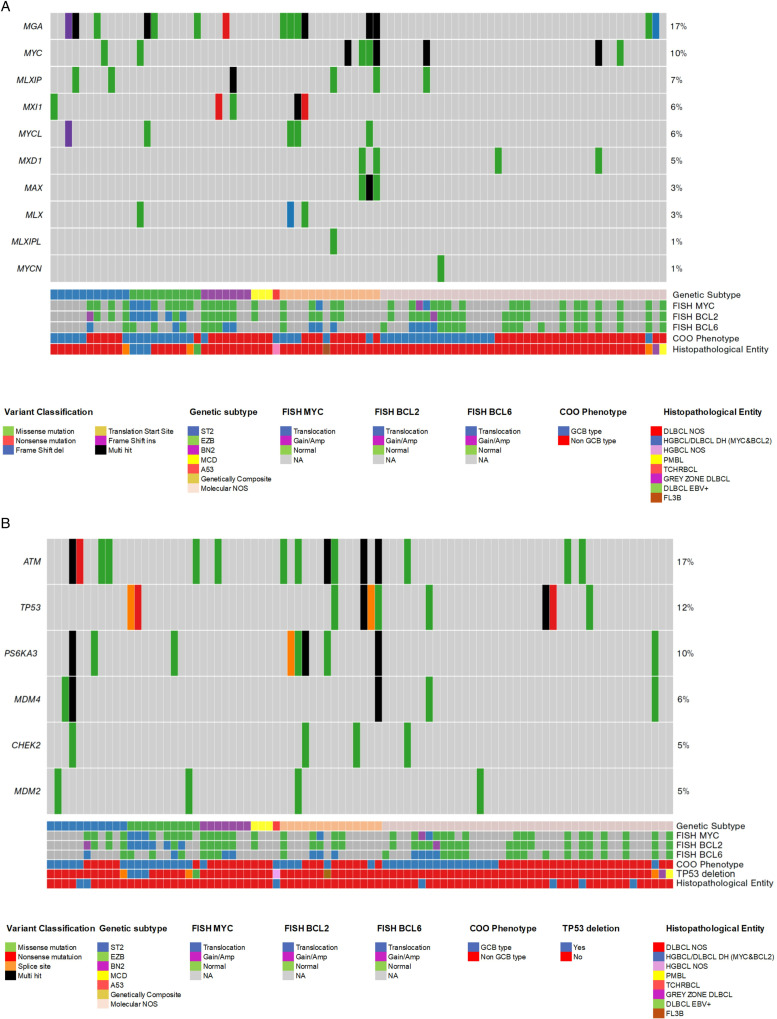
Analysis of MYC target pathways in DLBCL cases based on genetic subtype. MYC gene targets exhibited significant mutations in cases with specific genetic subtypes, in contrast to molecular NOS cases (Chi-square **p** <  0.001). Notably, there were no MYC target mutations observed in cases classified as pure MCD genetic subtype. B. Analysis of the TP53 pathway in DLBCL cases according to genetic subtype. TP53 heterozygous deletions were identified through predicted copy number alterations (CNAs) in seven out of 86 DLBCL cases (1 A53, 2 ST2, and 4 molecular NOS/Other). Somatic mutations in the TP53 pathway (including mutations in ATM, TP53, RPS6KA3, MDM4, CHECK2, and MDM2) were detected in 23 out of 46 (50%) genetically classified cases and in 9 out of 40 (22%) cases from the molecular NOS group. Somatic mutations in the TP53 pathway were significantly enriched in cases with defined genetic subtypes (Chi-square, **p** <  0.001). Reason: Improved clarity, readability, and technical accuracy while maintaining the original meaning.

We conducted a pathway analysis of MYC targets [14] to investigate the enrichment of mutations beyond the presence of MYC translocation. As illustrated in Fig 2, MYC targets were significantly mutated in cases with a specific genetic subtype compared to molecularly unclassified (NOS) cases (Chi-square test, p <  0.001). Notably, EZB and ST2, along with genetically composite cases, exhibited a high rate of mutations in MYC and MYC targets in a substantial proportion of cases, indicating activation of the MYC pathway independent of MYC translocation. In contrast, MCD type DLBCL cases demonstrated a lack of enrichment in the MYC targets pathway (Fig 2).

Anecdotally, one case classified as high-grade B-cell lymphoma not otherwise specified (NOS), based on its high-grade morphological features, was identified as molecular A53.

### MYD88 p.L265P mutation is preferentially found in DLBCL at extranodal sites but is not always associated with an MCD genetic profile

Out of 86 cases of diffuse large B-cell lymphoma (DLBCL), three exhibited a pure MCD genetic profile, representing 3% of the total. Additionally, four cases displayed a composite genetic makeup that included MCD features (specifically, ST2/MCD, EZB/MCD/ST2, BN2/MCD/ST2, and BN2/EZB/MCD/ST2). All cases with a pure MCD profile, as well as three composite cases featuring MCD, demonstrated a non-GC-B profile by IHC. In two of the three pure MCD cases, the MYD88 p.L265P mutation was identified using next-generation sequencing (NGS) and subsequently confirmed through allele-specific PCR (AS-PCR).

Indeed, the prevalence of the MYD88 p.L265P mutation was higher than that of MCD, with nine out of 86 cases (10%) exhibiting the MYD88 p.L265P mutation, as confirmed by orthogonal methods such as AS-PCR. Of these nine cases, two were classified as pure MCD by LymphGen. The remaining cases were classified as composite MCD (MCD/ST2, MCD/ST2/BN2, MCD/ST2/BN2/EZB) or categorized as Other/NOS (three cases). Therefore, the MYD88 p.L265P mutation alone was neither sufficient nor necessary to classify a case as pure MCD; concurrent genetic features are required for accurate classification. Conversely, six out of the nine cases with the MYD88 p.L265P mutation exhibited MCD features, whether pure or composite, according to LymphGen.

Consistent with previous observations, the MYD88 p.L265P mutation was more frequently identified in cases with extranodal biopsies[7,15–17], including those from the skin, oral cavity, testis, and breast in our series. In line with this, we evaluated an additional series of 71 extranodal DLBCL samples by AS-PCR and found a prevalence of the MYD88 p.L265P mutation of 45%, in sharp contrast to the less than 10% of nodal lymphoma cases that exhibited the mutation (see S2 Table in S1 File). Thus, the MYD88 p.L265P mutation is preferentially found in cases with extranodal involvement and is rare in purely nodal disease.

### *TP53* pathway enrichment across all DLBCL genetic subtypes

Due to the lack of TP53 ploidy data obtained from copy number variation (CNV) analysis, we used WES data to infer CNAs in an effort to identify the A53 genetic subgroup[2,3,7]. Additionally, we conducted a parallel analysis to identify the enrichment of TP53 pathway mutations based on the oncogenic pathway definitions provided by the Cancer Genome Atlas [14].

A single case of the A53 subtype was identified, which was classified as high-grade B-cell lymphoma NOS by histopathology. Consequently, cases exhibiting aneuploidy and TP53 inactivation were exceedingly rare in our cohort, accounting for only 5% to 10% of cases in previously published studies [7]. In contrast, other cluster definitions identify a greater number of cases with TP53 inactivation, such as those in the C2 cluster [3].

TP53 heterozygous deletions were identified through predicted CNAs in seven out of 86 DLBCL cases (one A53, two ST2 cases, and four classified as molecular NOS/Other). TP53 pathway, including mutations in ATM, TP53, RPS6KA3, MDM4, CHECK2, and MDM2, were detected in 23 out of 46 genetically classified cases, representing 50% of this group, and in nine out of 40 cases (22%) within the molecular NOS category. Notably, 17% of DLBCL cases exhibited mutations in ATM, while 11% displayed TP53 mutations, including hotspots in TP53 (p.Arg175His, COSV52661038, and p.Gly245Ser, COSV52661877). Concurrent mutations in both TP53 and ATM were observed in three cases.

Thus, somatic mutations in the TP53 pathway were significantly enriched in cases with a defined genetic subtype (Chi-square test, p <  0.001), suggesting a reduction in the transcriptional activity of TP53-related targets within the molecular NOS/Other subtype. Consistent with this concept, a case of A53 with TP53 aneuploidy exhibited no TP53 pathway somatic mutations.

### Genetic subtyping of Plasmablastic lymphoma shows ST2 features but significant genetic heterogeneity

Consistent with its frequent association with Epstein-Barr virus (EBV) infection and immune suppression, cases of plasmablastic lymphoma exhibited a significantly lower TMB when compared to other diffuse large B-cell lymphoma cases, regardless of genetic subtype. The median TMB for plasmablastic lymphoma cases was 6, while it was 17 for DLBCL cases (Mann-Whitney U test p-value =  0.003).

**Fig 3:**
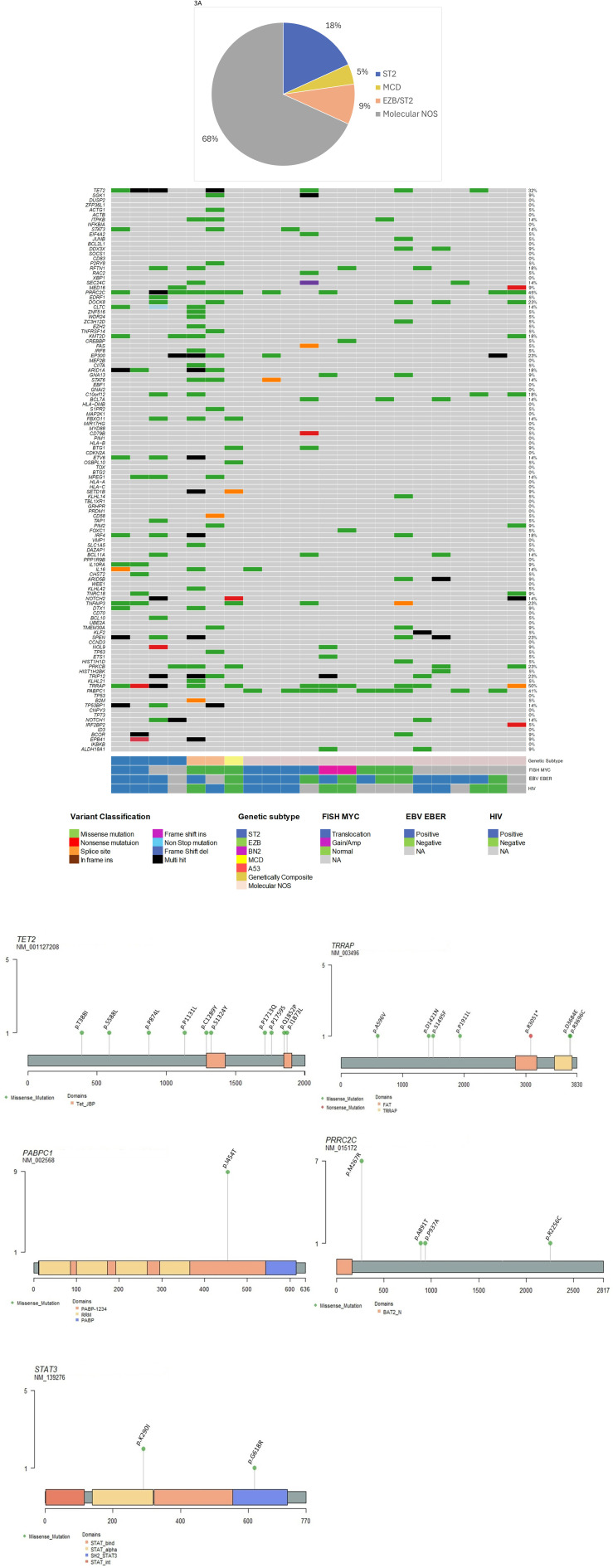
A. Prevalence of the ST2 genetic subtype in cases of plasmablastic lymphoma. B. Detailed analysis of LymphGen genetic features, highlighting an enrichment of genes associated with the ST2 subtype. C. Overview of protein localization for recurrent somatic mutations in TET2, TRRAP, PRRC2C, PABPC1, and STAT3.

Plasmablastic lymphomas exhibited ST2 genetic features in six out of 22 cases (27%), which included four cases with pure ST2 and two cases with a composite ST2/EZB profile (see Fig 3 and S4 Table in S1 File). We did not observe any association between EBV infection, HIV status, or MYC rearrangements and the ST2 genetics. The genes that were overrepresented in the ST2 cluster include TET2, STAT3, PRRC2C, DOCK8, and CLTC. The remaining cases of plasmablastic lymphoma were classified as molecular NOS/Other (68%), with one isolated case categorized as MCD.

**Fig 4:**
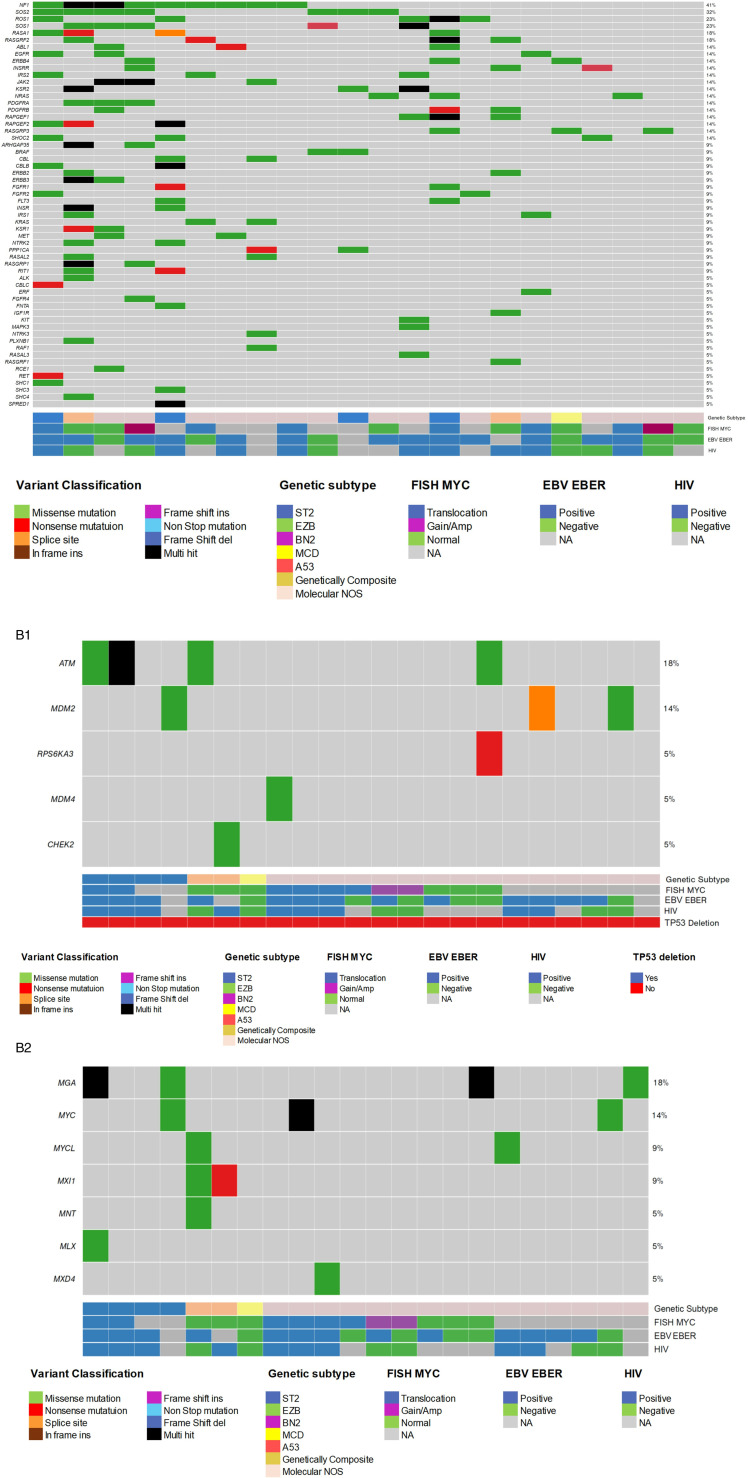
A. Enrichment of the RTK-RAS pathway in plasmablastic lymphoma. Note the recurrent mutations in the MAPK/ERK pathway, including NF1, BRAF, NRAS, KRAS, MAPK3, as well as NTRK2, NTRK3, and ROS1. B1. Analysis of TP53 target pathways in plasmablastic lymphoma cases according to genetic subtype. Structural aberrations of TP53 (heterozygous/homozygous loss) were absent in the twenty-two plasmablastic lymphoma cases. The TP53 pathway was significantly mutated in cases with ST2 genetics. B2. Analysis of MYC target pathways in plasmablastic lymphoma cases based on genetic subtype. MYC targets were recurrently mutated in this entity, particularly in cases with the ST2 and ST2/EZB genetic subtypes (4 out of 6 cases, 66%), regardless of the presence of MYC translocation as determined by FISH.

Pathway-focused analysis revealed an enrichment of mutated genes in the MAPK/ERK pathway within the plasmablastic lymphoma cohort, regardless of genetic subtype. Recurrent mutations were identified in NF1, BRAF, KRAS, NRAS, and MAPK3, as well as in the JAK/STAT pathway, including mutations in JAK2. Unexpectedly, recurrent mutations were also observed in other tyrosine kinase genes, such as EGFR, ERBB2, ERBB4, ALK, NTRK2/3, and ROS1, among others (Fig 4).

Pathway analysis revealed that MYC targets are frequently mutated in this entity, particularly in cases with the ST2 and ST2/EZB genetic subtypes (4 out of 6 cases, or 66%), regardless of the presence of MYC translocation (see Fig 4). Structural aberrations of TP53, including heterozygous and homozygous loss, were absent in the twenty-two cases of plasmablastic lymphoma. The TP53 pathway was significantly mutated in cases exhibiting ST2 genetic features, in contrast to those with molecular NOS/Other characteristics. TP53 pathway genes, such as ATM, MDM2, and CHEK2, were found to be mutated in 5 out of 6 ST2(/EZB) plasmablastic cases and in 3 out of 15 molecular NOS/Other plasmablastic cases.

## Discussion

In this study, we performed WES to characterize the genetic subtypes of a cohort comprising 108 cases of diverse large B cell lymphoma entities, primarily including mainly DLBCL NOS and plasmablastic lymphoma. Our findings indicate that genetic characterization based on WES and fusion data derived from conventional fluorescence in situ hybridization (FISH) testing, utilizing the LymphGen tool 2.0, is both feasible and effective, providing genetic subtype characterization in 55% of the cases. These results align with previous research [4,7] and demonstrate the consistency and reproducibility of the algorithm in identifying at least seven subtypes: EZB-MYCt, EZB, ST2, BN2, MCD, N1, and A53. Unlike previously published large validation cohorts [4], our study incorporated not only mutation-based data but also copy number alterations (CNAs) predicted from WES data, enabling us to identify rare A53 cases. Furthermore, a comparison of genetic subtype assignments based solely on somatic mutation data versus those incorporating both mutation data and CNAs prediction revealed eight cases with discordant results (8/108, 7%; see S1 Table 1 in S1 File), suggesting that the inclusion of CNAs is crucial for accurate subtype prediction.

Previous research on the genetic subtypes of diffuse large B-cell lymphoma (DLBCL) has primarily focused on the DLBCL not otherwise specified (NOS) category [3,7]. However, some data suggest that specific DLBCL entities may exhibit recurrent genetic features. A well-established example is the tendency of DLBCL to occur in extranodal sites, particularly in so-called immune-privileged sites (1), where it is more likely to present as the MCD genetic subtype[18–20]. Other specific DLBCL subtypes, such as high-grade B-cell lymphoma/DLBCL double-hit [21] and T-cell/histiocyte-rich B-cell lymphoma (TCHRBCL), may also display distinct genetic characteristics, such as EZB or ST2 [7], respectively. In this study, we have included a subset of cases with a histopathological diagnosis of plasmablastic lymphoma, which is characterized by recurrent mutations in the JAK/STAT and MAPK/ERK pathways [11,12], to investigate whether its genetic landscape aligns with any of the genetic subtypes described thus far.

Besides the detailed characterization of genetic features, genetic subtypes may prove useful for prognostication and therapy selection for patients at the time of diagnosis or disease progression [4,7,18,22]. Recent evidence has demonstrated that specific genetic subtypes exhibit differences in overall survival when patients are treated with conventional chemoimmunotherapy, with the N1, EZB-MYC, and MCD subtypes being variably associated with poor outcomes. Additionally, evidence from randomized phase II clinical trials supports the use of genetic subtype-guided chemoimmunotherapy, showing some benefit for patients with diffuse large B-cell lymphoma (DLBCL) [23,24].

Our results indicate that, overall, 55% of diffuse large B-cell lymphoma (DLBCL) cases were classified into specific genetic subtypes. Notably, DLBCL cases exhibited a marked dispersion in TMB scores within the series, demonstrating a non-normal distribution with slightly higher values than those reported in previously published cohorts [25,26]. These discrepancies may reflect differences in source material (formalin-fixed, paraffin-embedded genomic DNA) and variations in TMB calculation methodologies across studies. Importantly, DLBCL cases with a defined genetic subtype exhibited a significantly higher TMB compared to cases classified as molecular not otherwise specified (NOS) or other. Furthermore, significant differences in TMB were observed when comparing plasmablastic lymphoma cases to other DLBCL subtypes. This finding may suggest potential prognostic value, as higher TMB has been associated with improved survival in DLBCL patients treated with conventional chemoimmunotherapy [26].

ST2 genetic subtype is the most prevalent in our cohort of diffuse large B-cell lymphoma (DLBCL) cases, encompassing not only DLBCL NOS but also other rare DLBCL subtypes, such as T-cell/histiocyte-rich B-cell lymphoma (TCHRBCL) and plasmablastic lymphoma. Notably, after excluding specific DLBCL entities according to WHO classification (1), ST2 subtype was more frequent than the EZB subtype (13% vs. 7% in the DLBCL NOS subgroup) in our series. The observed differences in the prevalence of the most common genetic subtypes compared to previously published cohorts may be attributed to several factors, including restrictive diagnostic criteria and selection bias related to clinical trial enrollment criteria. The prognostic impact of the ST2 genetic subtype remains to be determined, along with potential precision medicine approaches that consider the prevalence of mutations in TET2 and the JAK/STAT signaling pathway in this subtype.

EZB subtype was primarily confined to GCB-DLBCL NOS (16%) and specific germinal center-derived DLBCL subtypes, such as T-cell Histiocyte-Rich B-cell Lymphoma and DLBCL/High-Grade B-cell Lymphoma with MYC and BCL2 dual hits. Notably, a single rare case of EBV + DLBCL with a non-GC-B phenotype exhibited EZB genetic characteristics. Importantly, two subsets were identified based on concomitant MYC translocation: EZB-MYCt and EZB-NOS, which demonstrate significant prognostic differences. In fact, all high-grade DLBCL cases with MYC and BCL2 translocations displayed these features, as previously described. In contrast, our cases of DLBCL NOS with MYC and BCL6 dual translocations exhibited diverse phenotypic and genetic characteristics (ST2, molecular NOS/Other), indicating a distinct origin beyond the germinal center dark zone.

In addition to EZB-MYC cases, we identified recurrent mutations in the MYC pathway in cases exhibiting EZB and ST2 genetic features, including composite cases. Most MYC mutations display characteristics consistent with aberrant somatic hypermutation, a hallmark of GC-B cell-derived lymphomas [27]. Notably, cases classified as the MCD subtype and those categorized as molecular NOS/Other demonstrated a scarcity of MYC target mutations.

(See Fig 2).

Consistent with previous observations, the MCD profile was identified in cases exhibiting a non-GC-B profile; however, we observed a relatively lower prevalence in our series compared to others [2,7]. Importantly, we demonstrated that the MYD88 p.L265P mutation alone is neither sufficient nor necessary to classify a case as pure MCD, indicating that additional genetic features are required. Indeed, the prevalence of the MYD88 p.L265P mutation was higher, with nine out of eighty-six (10%) cases exhibiting this mutation, as confirmed by orthogonal methods. Consequently, other genetic subtypes, such as composite MCD (MCD/ST2, MCD/ST2/BN2, MCD/ST2/BN2/EZB) or molecular NOS/Other, may also present with the MYD88 p.L265P mutation.

MYD88 p.L265P mutation was predominantly observed in cases with extranodal involvement and was infrequently found in purely nodal-based disease. The higher prevalence of the MYD88 L265P mutation and/or the MCD genetic profile in DLBCL of extranodal sites aligns well with previously published data. Notably, extranodal sites affected by lymphoma exhibiting these genetic features include not only immune-privileged sites, such as the central nervous system (CNS) and testis, as suggested by the current WHO classification (1), but also tumors arising in the breast, skin, nasal cavity, paranasal sinuses, adrenal glands, and soft tissues, as demonstrated by other studies[7,15,16,28]. Recent evidence suggests a memory B cell origin for these MCD/C5 lymphomas, which may account for their characteristic patterns of extranodal dissemination [20].

Intriguingly, we observed a higher-than-expected rate of cases classified with a composite genetic makeup (15%). These cases exhibited a greater tumor mutational burden (TMB) compared to the rest of the cohort, which may indicate significant intratumor heterogeneity in diffuse large B-cell lymphoma (DLBCL) tumors. Further research utilizing single-cell analysis may provide insights into the subclonal structure of the disease, potentially influencing tumor evolution.

Other genetic subtypes, such as BN2 (7 cases, 8%), characterized by NOTCH2 mutations and BCL6 translocations, were more prevalent in cases with a non-GC-B phenotype in our series. The BN2 profile has been reported to have a prevalence of approximately 15% in previous studies and is associated with intermediate outcomes.

Importantly, the analysis of the TP53 pathway revealed that both ATM and TP53 are somatically mutated in a significant proportion of diffuse large B-cell lymphoma (DLBCL) cases, approximately 20%, regardless of the genetic subtype. However, inactivation of TP53 was rare, with only one case of high-grade B-cell lymphoma NOS classified as the A53 subtype. Previous reports have documented the prevalence of both germline and somatic ATM mutations in DLBCL, suggesting a role for the ARF-TP53 pathway as a tumor suppressor in this disease [29], as well as the prognostic impact of TP53 hotspot mutations [30].

### Plasmablastic lymphoma exhibits ST2 genetic features in a substantial proportion of cases, along with widespread mutations in the JAK/STAT and MAPK/ERK pathways, as well as in druggable tyrosine kinases

Plasmablastic lymphoma genetic subtyping revealed a distinct profile comprising at least two subsets of cases. Genetic features associated with the ST2 subtype characterized a significant proportion of tumors (30%). These features include genes related to the activation of the JAK/STAT signaling pathway, such as STAT3, STAT6, and JAK2, which align well with previously published data (11, 12). Additionally, recurrent mutations in TET2, which is involved in the epigenetic regulation of gene expression, and TRRAP, a target of MYC transcriptional activation, were found to be enriched in this molecular subtype of plasmablastic lymphoma.

ST2 genetic subtype has previously been associated with other germinal center (GC) B cell-derived lymphomas, such as nodular lymphocyte-predominant Hodgkin lymphoma (NLPHL) and T-cell/histiocyte-rich large B-cell lymphoma (TCHRBCL), both of which typically exhibit a GC B cell phenotype. However, in our series, we identified ST2 features in six cases of plasmablastic lymphoma that demonstrated a complete loss of GC B cell markers. Additionally, in DLBCL NOS, eleven ST2 tumors exhibited both GCB and non-GCB phenotypes, suggesting that phenotypic heterogeneity within this group is greater than previously reported [7].

ST2 tumors exhibit a range of genetic characteristics, including features associated with germinal center B-cell (GCB) differentiation, such as truncating mutations in the TET2 gene [31]. Additionally, these tumors may harbor activating mutations in the JAK/STAT and NF-κB signaling pathways, including the inactivation of IκBα (NFKBIA) [7], a characteristic typically linked to activated B-cell (ABC) type diffuse large B-cell lymphoma (DLBCL), particularly in Epstein-Barr virus (EBV) positive cases [32]. It is possible that a subset of ST2 tumors represents B-cell neoplasms derived from GCB cells that have undergone terminal B-cell differentiation, driven by the sustained expression of plasma cell differentiation-related genes such as PRDM1/Blimp1 [33], and reinforced by the activation of the NF-κB and JAK/STAT pathways, among other potential oncogenic events.

A single case was classified as MCD due to the presence of MCD features in the absence of the MYD88 p.L265P mutation. However, approximately 70% of cases remained classified as molecular NOS/Other using the LymphGen algorithm. Our analysis of pathway enrichment revealed recurrent mutations in the MAPK/ERK pathway, including NF1, BRAF (p.V600E), NRAS, KRAS, MAPK3, and NTRK2/3. Recurrent mutations in other tyrosine kinase (TK) genes were also identified, including EGFR, ERBB2, ERBB4, ALK, and ROS1. Both MAPK/ERK pathway and TK mutations were observed in plasmablastic lymphoma cases, regardless of the genetic subtype. Mutations in the MAPK/ERK pathway, along with JAK/STAT pathway activation, have already been documented in plasmablastic lymphoma cases and may hold potential for targeted therapy and molecular diagnosis. These mutations result in JAK/STAT pathway overexpression, as indicated by transcriptional analysis [12,34] and immunohistochemical profiling [11]. Recently, unique activation patterns in the MAPK pathway have also been described in plasmablastic lymphoma, distinct from those observed in DLBCL, and involving the overexpression of NTRK genes (NTRK1 and NTRK2) [35]. These findings align with our mutational patterns, which include MAPK3 and NTRK2/3. Additional mutations affecting the RAS-MAPK pathway were found in BRAF, RAS genes, and NF1, corroborating previous reports [12].

Unexpectedly, recurrent mutations were also identified in other tyrosine kinase (TK) genes, including EGFR, ERBB2, ERBB4, ALK, and ROS1. Mutations in EGFR (p.V843I; COSV51767379) and ROS1 (p.R2269Q; COSV63862037) have been extensively documented in solid tumors, although they have been reported anecdotally in lymphoid neoplasms for ROS1 (COSMIC database). These mutations may also serve as potential markers for therapeutic intervention.

In summary, molecular subtyping of diffuse large B-cell lymphoma (DLBCL) using the LymphGen tool 2.0, along with whole exome sequencing (WES) and fluorescence in situ hybridization (FISH) data, provides precise molecular classification for approximately 55% of our cohort. The prevalence of genetic subtypes varies when compared to previously published studies, showing a higher prevalence of the ST2 genetic subtype in DLBCL NOS cases and a reduced prevalence of the MCD subtype. Clear associations between phenotypes and genetic subtypes (e.g., MCD with extranodal sites or EZB-MYC and high-grade B-cell lymphoma (HGBCL)/DLBCL with MYC and BCL2 double hits) suggest that these differences may be related to the inherent heterogeneity of DLBCL and the selection of cohorts. In addition to EZB-MYC cases, we identified recurrent mutations in the MYC pathway in cases exhibiting EZB and ST2 genetic features, including composite cases, indicating MYC pathway activation in a significant proportion of these instances.

The application of the LymphGen molecular classifier to plasmablastic lymphomas reveals intraspecific genetic diversity, with a significant proportion of ST2 tumors. Widespread somatic mutations indicate dysregulation of JAK/STAT, MAPK/ERK, and tyrosine kinase signaling pathways as potential markers for targeted therapy in this rare and aggressive lymphoma subtype.

## Methods

### Case selection

108 new cases were retrieved from the GELTAMO clinical trials (NCT01848132 and NCT2015-005390-21) as well as from retrospective multicentric series. The study and sample collection received approval from the local ethics committee (CEIC Cantabria, IRB code 2019.218). Research was conducted in accordance with relevant guidelines and regulations, adhering to the Declaration of Helsinki. Written informed consent was obtained from each patient at the time of clinical trial enrollment and/or prior to the inclusion of their samples in the Biobank facility. Patient clinical metadata was anonymized in compliance with internal Biobank regulations. Access to clinical metadata was granted during the research period, which commenced on December 1, 2019.

All cases were reviewed and diagnosed in accordance with the WHO classification of hematolymphoid neoplasms. A summary of the histopathological methods and pathological features of the cases can be found in the Supplementary Methods and S1 Table in S1 File.

### Next generation sequencing

DNA was extracted using conventional methods (see supplementary methods). A library containing whole exome regions was used to isolate the DNA for sequencing (SureSelect XT Human All Exon V6 (Agilent technologies). Sequencing on a NovaSeq 6000 instrument (Illumina, paired end, 2x100, mean 566Gb per FlowCell) was conducted at the National Genomic Analysis Center (CNAG, Barcelona, Spain).

### Sequencing data interpretation and reporting

Reads were mapped to human genome build hg19 with decoy sequences (hs37d5) using the BWA-MEM 0.7.17. Alignment files containing only properly paired, uniquely mapping reads. Without duplicates were processed using Picard [http://broadinstitute.github.io/picard/] to add read groups and remove duplicates. The Genome Analysis Tool Kit (GATK) was used for local realignment and base quality score recalibration. Somatic variant calling was done combining results from Mutect2 (from the GATK bundle) and Strelka2 [36]. In tumor only samples, just Mutect2 was used. Functional annotations were added using snpEff [37]. VCF conversion to MAF done using vcf2maf (Cyriac Kandoth. mskcc/vcf2maf: vcf2maf v1.6. (2020). https://doi.org/10.5281/zenodo.593251).

Single Nucleotide Polymorphims (SNPs) were filtered out based on the comparison of the Variant Allele Frequency (VAF) of the variant with the estimation of the amount of neoplastic cells by morphology and IHC, after search in dbSNP (http://www.ncbi.nlm.nih.gov/SNP/) and after comparison with available germline variants identified in each case and with a in house germline variants database. The COSMIC (http://cancer.sanger.ac.uk/cosmic) database was also checked in every case and the COSMIC Id was annotated. Selected variants were visualized using the Integrative Genomics Viewer (IGV).

CNAs were predicted with Exome Depth (30) with an internal database specifically constructed for the capture kit. Results were annotated with AnnotSV (31). Final tables were adapted to be used on NIH’s LymphGen Tool 2.0 (https://llmpp.nih.gov/lymphgen).

Tumor Mutational Burden was computed with pytmb [38], filtering out variants with Allelic Ratio <  0.05, MAF >  0.001, min Depth of 50, discarding non coding and synonymous variants and polymorphisms from gnomAD. Per library design we considered 60mB as the genome size.

Mann-Whitney U test, Shapiro-Milk and Chi-square tests were used to asses’ non-parametric data (i.e., TMB) as well as prevalence of genetic features among subgroups. Statistical significance was considered when two-sided p values < 0.05. Sankey plots were generated using SankeyMATIC.com.

## Supporting information

S1 File S1 Fig. A Summary of Variant classification in DLBCL NOS cases of specific genetic subtypes (51%, 38 cases). Note the higher mutational load in comparison with the molecular NOS cases. B Summary of Variant classification in DLBCL NOS of the molecular NOS/other subtype (49%, 37 case. **S2 Fig.** A complete representation of genetic subtype associated Lymphgen features found on each case of DLBCL. Genetic subtype prediction, FISH results for MYC, BCL2 and BCL6, Histopathological subtype and cell of origin phenotype are shown. **S3 Fig.** Oncogenic signaling pathway analysis focusing on the RTK/RAS pathway in molecular NOS/Other DLBCL cases. Somatic mutations in genes such as ROS1 (7 cases) were prevalent in these cases. Mutations in MAPK/ERK pathway were absent with isolated cases showing NF1 (4 cases), BRAF (1 case) and NRAs (1 case) somatic mutations. B. Detail of gene locations for mutations in ROS1, NF1 and ALK. **S4 Fig.** TP53 somatic mutations in DLBCL cases. Detailed protein location of TP53 gene mutations are shown. Note the presence of hotspot mutations such as (p.Arg175His COSV52661038 and p.Gly245Ser, COSV52661877). **S5 Fig.** Detailed protein location of recurrent mutations in MAPK/ERK pathway genes, EGFR and ROS1 in plasmablastic lymphoma. **S6 Fig.** druggable categories in plasmablastic lymphoma according to mutational landscape. **S1 Table.** Summary of pathological features of the cohort of cases. Data regarding histopathological diagnosis according to current WHO classification, phenotype based on immunohistochemistry and/or gene expression profiling (LymphC2x), MYD88 L265P mutation status by allele-specific PCR and/or NGS in the tumor biopsy, FISH results, TP53 CNAs prediction, Lymphgen 2.0 genetic subtype predictions, EBV-EBER by in situ hybridization in tumor biopsy and HIV status are shown. **S2 Table.** MYD88L265P mutation status detected by AS-PCR in an independent series of 112 DLBCL samples, including seventy-one extranodal DLBCL. The prevalence of MYD88L265P mutation in extranodal samples was 45%, in contrast with <10% of nodal Lymphoma cases. **S3 Table.** Summary genetic subtype associated Lymphgen features found on each case of DLBCL. **S4 Table.** Summary of Lymphgen features found on each case of Plasmablastic Lymphoma. **S5 Table.** List of somatic variants identified in 108 cases. This list includes all type of variants (synonymous, intronic, missense, nonsense, splice site, frame-shift deletions, frame-shift insertions, in frame deletions, in-frame insertions, translation start site, non-stop mutations) with a DP superior or equal to 50 reads. For evaluation of complete raw data for each case, including germline DNA sequencing when available, please consider to log in the EGA repository (https://ega-archive.org/studies/EGAS50000000371). Request for data access may be referred directly to the Data Access Committee: https://ega-archive.org/dacs/EGAC50000000261
(ZIP)
