# Supplementary material for: Genetic subtyping by Whole Exome Sequencing across Diffuse Large B Cell Lymphoma and Plasmablastic Lymphoma
Source: PLoS One. 2025 Mar 11;20(3):e0318689. doi: 10.1371/journal.pone.0318689 (PMC11896070; doi:10.1371/journal.pone.0318689)
Supplement: S1 File — Note the higher mutational load in comparison with the molecular NOS cases. B Summary of Variant classification in DLBCL NOS of the molecular NOS/other subtype (49%, 37 case. S2 Fig. A complete representation of genetic subtype associated Lymphgen features found on each case of DLBCL. Genetic subtype prediction, FISH results for MYC, BCL2 and BCL6, Histopathological subtype and cell of origin phenotype are shown. S3 Fig. Oncogenic signaling pathway analysis focusing on the RTK/RAS pathway in molecular NOS/Other DLBCL cases. Somatic mutations in genes such as ROS1 (7 cases) were prevalent in these cases. Mutations in MAPK/ERK pathway were absent with isolated cases showing NF1 (4 cases), BRAF (1 case) and NRAs (1 case) somatic mutations. B. Detail of gene locations for mutations in ROS1, NF1 and ALK. S4 Fig. TP53 somatic mutations in DLBCL cases. Detailed protein location of TP53 gene mutations are shown. Note the presence of hotspot mutations such as (p.Arg175His COSV52661038 and p.Gly245Ser, COSV52661877). S5 Fig. Detailed protein location of recurrent mutations in MAPK/ERK pathway genes, EGFR and ROS1 in plasmablastic lymphoma. S6 Fig. druggable categories in plasmablastic lymphoma according to mutational landscape. S1 Table. Summary of pathological features of the cohort of cases. Data regarding histopathological diagnosis according to current WHO classification, phenotype based on immunohistochemistry and/or gene expression profiling (LymphC2x), MYD88 L265P mutation status by allele-specific PCR and/or NGS in the tumor biopsy, FISH results, TP53 CNAs prediction, Lymphgen 2.0 genetic subtype predictions, EBV-EBER by in situ hybridization in tumor biopsy and HIV status are shown. S2 Table. MYD88L265P mutation status detected by AS-PCR in an independent series of 112 DLBCL samples, including seventy-one extranodal DLBCL. The prevalence of MYD88L265P mutation in extranodal samples was 45%, in contrast with <10% of nodal Lymphoma cases. S3 Table. Summary genetic subt [file pone.0318689.s001.zip › supplementary material/FIG S3A. OncoPlot RTKRAS DLBCL Other.pdf]

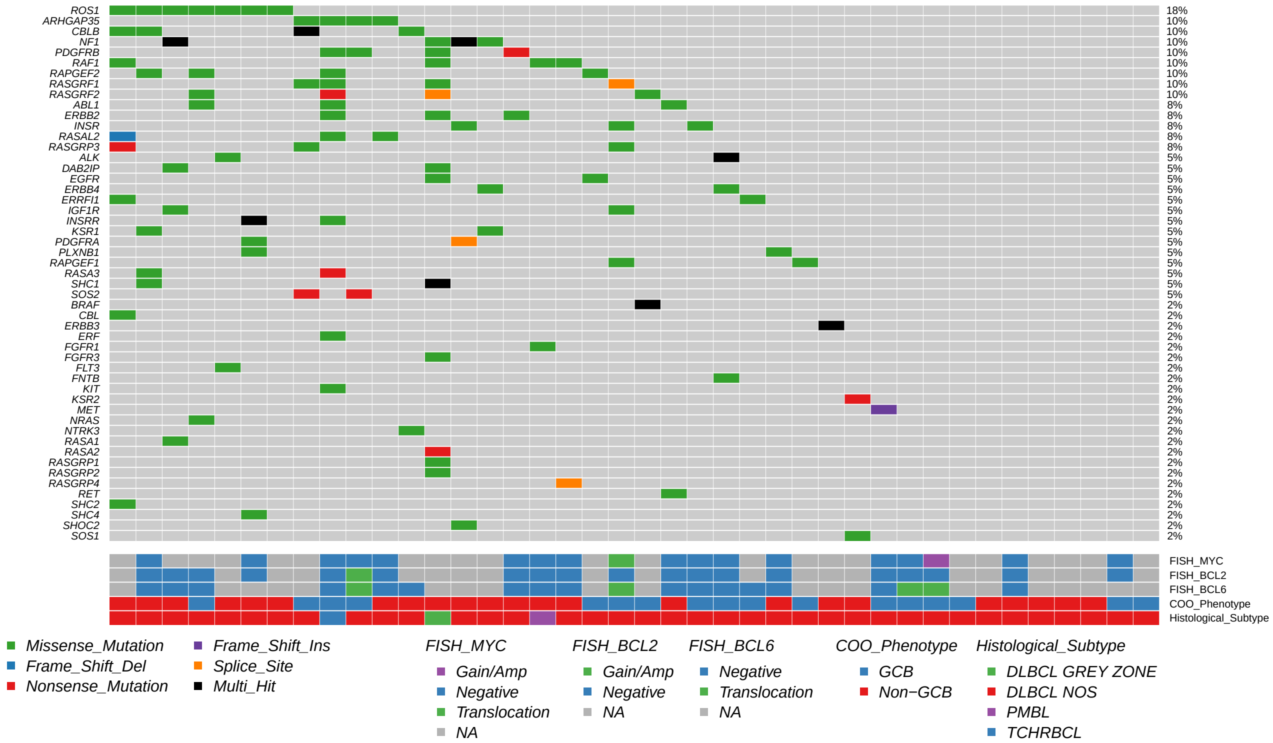
ROS1 18%
ARHGAP35 10%
CBLB 10%
NF1 10%
PDGFRB 10%
RAF1 10%
RAPGEF2 10%
RASGRF1 10%
RASGRF2 10%
ABL1 8%
ERBB2 8%
INSR 8%
RASAL2 8%
RASGRP3 8%
ALK 5%
DAB2IP 5%
EGFR 5%
ERBB4 5%
ERRFI1 5%
IGF1R 5%
INSRR 5%
KSR1 5%
PDGFRA 5%
PLXNB1 5%
RAPGEF1 5%
RASA3 5%
SHC1 5%
SOS2 5%
BRAF 2%
CBL 2%
ERBB3 2%
ERF 2%
FGFR1 2%
FGFR3 2%
FLT3 2%
FNTB 2%
KIT 2%
KSR2 2%
MET 2%
NRAS 2%
NTRK3 2%
RASA1 2%
RASA2 2%
RASGRP1 2%
RASGRP2 2%
RASGRP4 2%
RET 2%
SHC2 2%
SHC4 2%
SHOC2 2%
SOS1 2%
FISH_MYC
FISH_BCL2
FISH_BCL6
COO_Phenotype
Histological_Subtype
Missense_Mutation
Frame_Shift_Del
Nonsense_Mutation
Frame_Shift_Ins
Splice_Site
Multi_Hit
FISH_MYC
Gain/Amp
Negative
Translocation
NA
FISH_BCL2
Gain/Amp
Negative
NA
FISH_BCL6
Negative
Translocation
NA
COO_Phenotype
GCB
Non−GCB
Histological_Subtype
DLBCL GREY ZONE
DLBCL NOS
PMBL
TCHRBCL
