# Supplementary material for: Genetic subtyping by Whole Exome Sequencing across Diffuse Large B Cell Lymphoma and Plasmablastic Lymphoma
Source: PLoS One. 2025 Mar 11;20(3):e0318689. doi: 10.1371/journal.pone.0318689 (PMC11896070; doi:10.1371/journal.pone.0318689)
Supplement: S1 File — Note the higher mutational load in comparison with the molecular NOS cases. B Summary of Variant classification in DLBCL NOS of the molecular NOS/other subtype (49%, 37 case. S2 Fig. A complete representation of genetic subtype associated Lymphgen features found on each case of DLBCL. Genetic subtype prediction, FISH results for MYC, BCL2 and BCL6, Histopathological subtype and cell of origin phenotype are shown. S3 Fig. Oncogenic signaling pathway analysis focusing on the RTK/RAS pathway in molecular NOS/Other DLBCL cases. Somatic mutations in genes such as ROS1 (7 cases) were prevalent in these cases. Mutations in MAPK/ERK pathway were absent with isolated cases showing NF1 (4 cases), BRAF (1 case) and NRAs (1 case) somatic mutations. B. Detail of gene locations for mutations in ROS1, NF1 and ALK. S4 Fig. TP53 somatic mutations in DLBCL cases. Detailed protein location of TP53 gene mutations are shown. Note the presence of hotspot mutations such as (p.Arg175His COSV52661038 and p.Gly245Ser, COSV52661877). S5 Fig. Detailed protein location of recurrent mutations in MAPK/ERK pathway genes, EGFR and ROS1 in plasmablastic lymphoma. S6 Fig. druggable categories in plasmablastic lymphoma according to mutational landscape. S1 Table. Summary of pathological features of the cohort of cases. Data regarding histopathological diagnosis according to current WHO classification, phenotype based on immunohistochemistry and/or gene expression profiling (LymphC2x), MYD88 L265P mutation status by allele-specific PCR and/or NGS in the tumor biopsy, FISH results, TP53 CNAs prediction, Lymphgen 2.0 genetic subtype predictions, EBV-EBER by in situ hybridization in tumor biopsy and HIV status are shown. S2 Table. MYD88L265P mutation status detected by AS-PCR in an independent series of 112 DLBCL samples, including seventy-one extranodal DLBCL. The prevalence of MYD88L265P mutation in extranodal samples was 45%, in contrast with <10% of nodal Lymphoma cases. S3 Table. Summary genetic subt [file pone.0318689.s001.zip › supplementary material/supplementary material_figure and table legends.docx]

Supplementary Data

Supplementary Methods

**Case Selection**

108 new cases were obtained from the GELTAMO clinical trials (NCT01848132 and NCT2015-005390-21) as well as from a retrospective multicentric series. This cohort includes twenty-two cases of plasmablastic lymphoma sourced from our collection of cases submitted for second opinions in our pathology reference service at Valdecilla.

The study and sample collection were approved by the local ethics committee (CEIC Cantabria, IRB code 2016.168) and comply with the Declaration of Helsinki. All cases were reviewed and diagnosed according to the WHO classification of Hematolymphoid Neoplasms. A summary of the pathological features of the cases has been recorded and is available in Supplementary Table 1.

**Immunohistochemistry and in situ hybridization.**

Immunohistochemical reactions were conducted using standard automated procedures on DAKO Autostainer and Omnis automated platforms.

The following antibodies were utilized: CD20 (DAKO, ready-to-use), PAX-5 (DAKO, ready-to-use), CD138 (DAKO, ready-to-use), CD38. (Leica, 1:200), IRF4/MUM-1 (DAKO, ready-to-use); Blimp-1 (CNIO, 1:5); Kappa (DAKO, ready-to-use); Lambda (DAKO, ready-to-use); BCL-6 (DAKO, ready-to-use), BCL-2 (DAKO, ready-to-use), CD10 (DAKO, ready-to-use), KI67 (DAKO, ready-to-use), CMYC (Abcam, dilution 1:50), HHV-8 (Novus Biologicals, dilution 1:10), EBV-LMP1 (DAKO, ready-to-use), CD30 (DAKO, ready-to-use), ALK (DAKO, ready-to-use), and p53 (DAKO, ready-to-use).

EBV-EBER was performed using CISH. The staining was considered positive when 80% or more of the large atypical cells exhibited positivity.

Fluorescent in situ hybridization (FISH) for the detection of MYC, BCL2, and BCL6 rearrangements was performed using a dual-color break-apart rearrangement probe set specific for the MYC gene locus on chromosome 8q24.21 (Abbott Molecular), the BCL2 gene locus on chromosome 18q21.33 (Abbott Molecular), and the BCL6 gene locus on chromosome 3q27.3 (Abbott Molecular). A minimum of 10% of cells displaying a break-apart signal was required for a case to be classified as positive for rearrangements. Additionally, at least 15% of cells with extra copies of the gene were necessary to identify a case as positive for copy number gains of the gene.

**DNA extraction for sequencing analysis.**

DNA was extracted from formalin fixed paraffin embedded samples using the PicoPure™ DNA Isolation Kit (ThermoFisher Scientific). A limited number of DNA samples derived from available ctDNA samples (QIAamp Circulating Nucleic Acid Kit, Qiagen). DNA was quantified by Qbit fluorometer (ThermoFisher Scientific). All samples subjected to NGS analysis were required to have >50% of neoplastic cells as identified by morphology (H&E) in the available diagnostic FFPE material.

**Next Generation Sequencing.**

A library containing whole exome regions was used to isolate the DNA for sequencing (SureSelect XT  Human All Exon V6 (Agilent technologies)). Covaris S2 was used to fragment DNA and quality control was performed with Bioanalyzer 2100 (Agilent).

Sequencing on a NovaSeq 6000 instrument (Illumina, paired end, 2x100, mean 566Gb per FlowCell) at the National Genomic Analysis Center (CNAG, Barcelona, Spain) was performed.

**Sequencing data interpretation and reporting.**

Reads were mapped to human genome build hg19 with decoy sequences (hs37d5) using the

BWA-MEM 0.7.17. Alignment files containing only properly paired, uniquely mapping reads

without duplicates were processed using Picard [http://broadinstitute.github.io/picard/] to add

read groups and to remove duplicates. The Genome Analysis Tool Kit (GATK) was used for local

realignment and base quality score recalibration. Somatic variant calling was done combining results from Mutect2 (from the GATK bundle) and Strelka2 (33). In tumor only samples, just Mutect2 was used. Functional annotations were added using snpEff(34). VCF conversion to MAF done using vcf2maf (Cyriac Kandoth. mskcc/vcf2maf: vcf2maf v1.6. (2020). doi:10.5281/zenodo.593251).

Single Nucleotide Polymorphims (SNPs) were filtered out based on the comparison of the Variant Allele Frequency (VAF) of the variant with the estimation of the amount of neoplastic cells by morphology and IHC, after search in dbSNP (http://www.ncbi.nlm.nih.gov/SNP/) and after comparison with available germline variants identified in each case and with a in house germline variants database. The COSMIC (http://cancer.sanger.ac.uk/cosmic) database was also checked in every case and the COSMIC Id was annotated. Selected variants were visualized using the Integrative Genomics Viewer (IGV).

CNAs were predicted with Exome Depth(30) with an internal database specifically constructed

for the capture kit. Results were annotated with AnnotSV (31). Final tables were adapted to be

used on NIH's LymphGen Tool 2.0 (<https://llmpp.nih.gov/lymphgen>).

Tumor Mutational Burden was computed with pytmb(35), filtering out variants with Allelic Ratio < 0.05, MAF > 0.001, min Depth of 50, discarding non coding and synonymous variants and polymorphisms from gnomAD.

**Lymph gen 2.0 tool analysis, descriptive statistical analysis and cluster representation**

Required input files were generated based on NGS data, including sample annotation file, mutation flat file, mutation gene list file, copy number flat file, Copy Number Gene List file, Arm Flat file. Available fusion data for BCL2 and BCL6 derived from conventional FISH annalysis were used to classify the cases according to Lymphgen v2 (<https://llmpp.nih.gov/lymphgen>). HETLOSS and GAIN only flag was defined for copy number class prediction, according to available CN data.

XLSTAT Biomed software (version 19.4) was used for statistical analysis. Descriptive statistics were performed. The package Maftools from R Bioconductor (R version 4.0.3, maftools version 2.6.05) (Mayakonda A, Lin DC, Assenov Y, Plass C, Koeffler HP. 2018. Maftools: efficient and comprehensive analysis of somatic variants in cancer. Genome Resarch. PMID: 30341162) was used to summarize, analyze and Visualize data, including variant type and classification, oncoplots, Lollipop plots for amino acid changes, Drug-Gene Interactions and Oncogenic Signaling Pathways based on on the Cancer genome Atlas oncogenic pathway definition Sanchez-Vega F, Mina M, Armenia J, Chatila WK, Luna A, La KC, et al. Oncogenic Signaling Pathways in The Cancer Genome Atlas. Cell. 2018;173(2):321-37.e10.

**LymphC2x RNA expression annalysis.**

Digital quantitative PCR (LymphC2x, Nanostring®) was performed at the external laboratory (Pathology Department, Hospital Clínic, Barcelona, Spain) to classify patients as ABC, GCB, or unclassified. Digital quantitaive PCR was performed using FFPE derived RNA as a template.

**Supplementary Tables and Figures legends**

**Supplementary figure 1.** A Summary of Variant classification in DLBCL NOS cases of specific genetic subtypes (51%, 38 cases). Note the higher mutational load in comparison with the molecular NOS cases. B Summary of Variant classification in DLBCL NOS of the molecular NOS/other subtype (49%, 37 cases.

**Supplementary Figure 2. A complete representation of genetic subtype associated Lymphgen features found on each case of DLBCL.** Genetic subtype prediction, FISH results for MYC, BCL2 and BCL6, Histopathological subtype and cell of origin phenotype are shown.

**Supplementary Figure 3. Oncogenic signaling pathway analysis focusing on the RTK/RAS pathway in molecular NOS/Other DLBCL cases**. Somatic mutations in genes such as ROS1 (7 cases) were prevalent in these cases. Mutations in MAPK/ERK pathway were absent with isolated cases showing NF1 (4 cases), BRAF (1 case) and NRAs (1 case) somatic mutations. B. Detail of gene locations for mutations in ROS1, NF1 and ALK.

**Supplementary Figure 4.** TP53 somatic mutations in DLBCL cases. Detailed protein location of TP53 gene mutations are shown. Note the presence of hotspot mutations such as (p.Arg175His COSV52661038 and p.Gly245Ser, COSV52661877).

**Supplementary figure 5. Detailed protein location of recurrent mutations in MAPK/ERK pathway genes, EGFR and ROS1 in plasmablastic lymphoma.**

**Supplementary figure 6: druggable categories in plasmablastic lymphoma according to mutational landscape.**

**Supplementary Table 1. Summary of pathological features of the cohort of cases.** Data regarding histopathological diagnosis according to current WHO classification, phenotype based on immunohistochemistry and/or gene expression profiling (LymphC2x), MYD88 L265P mutation status by allele-specific PCR and/or NGS in the tumor biopsy, FISH results, TP53 CNAs prediction, Lymphgen 2.0 genetic subtype predictions, EBV-EBER by in situ hybridization in tumor biopsy and HIV status are shown.

**Supplementary Table 2. MYD88L265P mutation status detected by AS-PCR in an independent series of 112 DLBCL samples, including seventy-one extranodal DLBCL.** The prevalence of MYD88L265P mutation in extranodal samples was 45%, in contrast with <10% of nodal Lymphoma cases.

**Supplementary table 3. Summary genetic subtype associated Lymphgen features found on each case of DLBCL.**

**Supplementary table 4. Summary of Lymphgen features found on each case of Plasmablastic Lymphoma.**

**Supplementary table 5. List of somatic variants identified in 108 cases.** This list includes all type of variants (synonymous, intronic, missense, nonsense, splice site, frame-shift deletions, frame-shift insertions, in frame deletions, in-frame insertions, translation start site, non-stop mutations) with a DP superior or equal to 50 reads. For evaluation of complete raw data for each case, including germline DNA sequencing when available, please consider to log in the EGA repository (<https://ega-archive.org/studies/EGAS50000000371>). Request for data access may be referred directly to the Data Access Committee: https://ega-archive.org/dacs/EGAC50000000261.
